# Life-Course Pathways to Cognitive Aging: The Significance of Intellectual Stimulation in the Form of Education and Occupation for Public Policy and Prevention Plans

**DOI:** 10.3389/fpsyt.2021.719609

**Published:** 2021-07-22

**Authors:** Francisca S. Rodriguez

**Affiliations:** ^1^German Center for Neurodegenerative Diseases (DZNE), RG Psychosocial Epidemiology and Public Health, Greifswald, Germany; ^2^Center for Cognitive Science, University of Kaiserslautern, Kaiserslautern, Germany; ^3^Institute of Social Medicine, Occupational Medicine and Public Health, University of Leipzig, Leipzig, Germany

**Keywords:** old age, dementia, cognitive decline, prevention, life-course

## Introduction

With life expectancy increasing, many of us can hope to live a long life. However, older age comes with a higher risk for a variety of chronic health conditions such as for cardiovascular, respiratory, musculoskeletal, and neurological diseases that can make it difficult to enjoy getting older (1). One of the most prevalent neurological diseases in old age is dementia. Its prevalence increases with older age. While in the 70–74 years' age group, the prevalence of dementia is only around 3%, already about 12% in the 80–84 years' age group and more than 20% in the 90+ years' age group have dementia (2). Accordingly, the number of people with dementia will be increasing, as populations grow older. Estimations suggest that the prevalence worldwide is around 50 million people with nearly 10 million new cases every year (3). Therefore, dementia does not only affect the individual but society as a whole.

For more than a century, research on dementia has focused on biological aspects of cognitive disorders such as brain imaging and biomarkers (4). However, the neurobiological changes in the aging brain (5) are influenced by behavior and by the environment (6). More and more ongoing research reveals the relevance of social determinants and lifestyle factors for the risk of developing dementia (7).

## Life-Course Approach

Taking a life-course approach to cognitive health, which explicitly includes social and lifestyle factors, can contribute to a better prevention. Research taking this approach aims at understanding how biological, behavioral, social, and psychological exposures are important for health in later life (8). The Life Course Health Development (LCHD) framework considers health to be the result of multiple interactions between the environment and the bio-behavioral regulatory systems of the human body (9). From that perspective, health trajectories are the cumulative product of risks and protective factors (9). Disparities in health between people of varying socioeconomic status are the result of psychological and social influences e.g., stress, social exclusion, working conditions, unemployment, healthy food etc. (10). In that sense, healthy aging is a multifaceted phenomenon that can only be understood by holistically studying the complexity of social and lifestyle factors in the context of aging (11). The “biological capital” that an individual has acquired in their lifetime determines how long they remain above a critical threshold for adverse health outcomes (12). This is a perspective adopted by the World Health Organization (WHO), which states that “Healthy aging is about creating the environments and opportunities that enable people to be and do what they value throughout their lives” (13).

Cognitive health also depends on multiple determinants and their dynamics throughout the life-course. The Cognitive Health Environment Life Course Model (CHELM) summarizes lifestyle factors that influence cognitive health in old age (14). Lifestyle can positively or negatively moderate disease risk via neuropathological changes (14). Indeed, lifestyle can affect cognitive health to a great extent. Estimates suggest that up to 3 million dementia cases worldwide could be prevented if it was possible to reduce known risk factors by 25% (15). This type of evidence calls for modification of public health policies to prevent cognitive decline and dementia (16).

## The Role of Intellectual Stimulation in the Form of Education and Occupation

Ever since researchers started investigating dementia, they observed that people with higher education develop dementia less frequently and express less severe dementia symptoms in comparison to low-educated people with the same level of Alzheimer's disease (AD) pathology in the brain (17). In research, this effect is called “cognitive reserve” (18). The interesting aspect is that a simple socio-environmental factor can possibly provide such resilience against a biologically devastating disease. Health status or income do not explain this resilience (19, 20). Rather, intellectual stimulation across the lifespan seems to be the central factor in this cognitive reserve effect. Moreover, intellectual stimulation by the environment can help the brain to remain healthy for longer by a process called “brain maintenance” (21). Indications on how intellectual stimulation leads to better brain maintenance come from rodent studies. Relevant studies have shown that exposure to enriched housing (i.e., an enriched environment) compared to standard housing conditions can protect against neurodegenerative diseases (22). Exposure to enriched environments seems to up-regulate synaptogenesis and synaptic plasticity and to activate epigenetic mechanisms (22). In this way, the intellectual stimulation provided by enriched environments can improve brain maintenance.

Evidence from longitudinal studies largely confirms that intellectually stimulating activities over the life-course protect cognitive health in old age. As summarized in the Life Course Model of Cognitive Reserve (LCMCR), an intellectually engaged lifestyle builds up a “cognitive reserve” that protects cognitive health in old age (23). The model highlights that all stages across the life-course—starting from cognitive development in childhood to hobbies in later life—can modify cognitive health (23). Results from the majority of longitudinal studies confirm that higher education is associated with benefits for cognitive functioning in later life (24). The socioeconomic attainment related to education has little impact on dementia risk, even in minority populations (20). Instead, it appears that the difference in the number of years of education can explain ethnic and racial disparities in dementia prevalence (20). However, high education does not prevent cognitive decline. Instead, it seems that highly educated individuals have a seven to 8 year period of only limited cognitive decline before their cognitive abilities deteriorate to the same extent as they do in low-educated individuals (25). Biological evidence reinforces the idea that education does not prevent dementia, as higher education does not seem to be associated with a reduction in AD biomarkers of Alzheimer's disease (26). Rather, studies have pointed out that higher education is associated with a higher brain network efficiency, even in the presence of such biomarkers (tau pathology) in the brain (26). This may reflect compensatory mechanisms in the brains of highly educated people.

In addition, evidence from longitudinal studies also accentuates the relevance of intellectual stimulation at work. Higher mental demands in the occupational context are shown to be associated with a lower dementia risk (27). Specifically, people who were working in jobs that challenge executive cognitive abilities have a lower dementia risk (27) and slower cognitive decline (28). Similar observations are made in people who were working in jobs with complex information processing (29). Importantly, these and other studies have shown that not all types of mental demands at work provide this beneficial effect. The complexity of information processing required appears to be critical (29). Based on Wickens's model of human information processing, tasks that are more complex require more attentional resources, a higher workload, and more integration of long-term memories (30). It is possible that this simultaneous activation of several cognitive resources will help to protect the brain longer.

Yet, the exact mechanisms remain unclear. On a cognitive level, people with high education seem to have superior semantic memory and executive cognitive abilities (31). These abilities provide them with a larger knowledge base and skills that constitute an advantage in terms of employing memory compensation strategies (32). Observations from computerized cognitive training show structural and functional brain changes related to the domain that was trained (33). Accordingly, a training in semantic memory and executive cognitive abilities might delay cognitive decline. Moreover, we would assume that maximizing the complexity of information processing in cognitive training tools might maximize training effects. However, differentiated evidence on this is not yet available.

## Benefits for High-Risk Groups

People with a greater risk for dementia can benefit from intellectual stimulation throughout the life-course. Genetically, people who carry an Apolipoprotein E (APOE) e4 allele are considered to be a high-risk group for dementia (34). Research has shown that associations between lifestyle factors and cognitive functioning are generally similar in APOE e4 allele carriers and non-carriers (35, 36). Accordingly, a preventive lifestyle approach is also beneficial for this group. Increasing education, in particular, may be relevant to this high-risk group as low education comes with a higher dementia risk in APOE e4 allele carriers than in non-carriers (37). Looking specifically at low-educated people, findings demonstrate that low-educated APOE e4 allele carriers had a larger decrease in the metabolism in medial-temporal and prefrontal areas in old age than low-educated non-carriers (38). Low education might thus be inflating dementia risk among people with the high-risk variant of the APOE e4 gene.

Another important risk factor for dementia is social isolation (39, 40). Evidence suggests that intellectual stimulation may also be beneficial for the socially isolated high-risk group. Regarding education, research findings indicate that the association between social isolation and memory performance is stronger among people with low education than high education (41). These findings suggest that low-educated individuals experience greater adverse effects from isolation. Education might thus shield against the negative impact of social isolation. Similar findings are made regarding people with high mental demands at work, who experienced less adverse effects from social isolation on their cognitive functioning than people with low mental demands at work (41, 42). The cause of this effect is yet unknown. Yet, the evidence suggests that intellectual stimulation is clearly advisable for people who live socially isolated.

## Prevention Strategies

Intellectual stimulation in the form of education, occupation, or other cognitive activities is associated with important protective effects for cognitive health. Accordingly, it should be integrated in effective prevention strategy programs. First initiatives to offer cognitive training programs have been created, e.g., the Advanced Cognitive Training for Independent and Vital Elderly (ACTIVE) (43) and the Improvement in Memory with Plasticity-based Adaptive Cognitive Training (IMPACT) Study (44). In addition, the first multi-dimensional training programs (including cognitive training, diet, exercise, and vascular risk monitoring), such as the Finnish Geriatric Intervention Study to Prevent Cognitive Impairment and Disability (FINGER, 45) are being implemented. As far as publicly known, FINGER was the first study of this kind. It started in 2009 and offered a 2-year intervention for people at a high risk for developing dementia. After the 2 years, performance in all cognitive sub-domains was improved (45). By today, FINGER has inspired further large multi-domain intervention studies worldwide, which are connected in the World-Wide FINGERS, the first global network of multi-domain lifestyle intervention trials (https://www.alz.org/wwfingers/overview.asp).

WHO guidelines likewise recommend a proactive multi-dimensional approach to risk reduction of cognitive decline and dementia (46). Recommendations comprise physical activity, nutritional interventions, social activity, tobacco and alcohol abuse cessation, and better management of chronic diseases (46). Further, the WHO report includes a conditional recommendation for cognitive training for people with mild cognitive impairments. Even though many studies show significant effects of cognitive training, high quality evidence is still lacking—a conclusion also reached by the Lancet commission on dementia prevention (47). Irrespective of any benefit from short-term trainings, it is important to acknowledge the greater benefit of cumulative exposure to intellectual stimulation in all of its form throughout the life-course (48), which so far has been underestimated.

While the above-mentioned studies constitute important progress, major problems persist: First, the programs typically end when the respective study ends. Second, only a limited number of people in the population had the chance to benefit from the program. Third, the infrastructure necessary to implement such programs on a population scale is, as of now, rarely available. Table 1 summarizes a number of challenges that need to be addressed. Given the potential of intellectual stimulation to shape the expression of symptoms at each disease level, it should be a persistent part of secondary and tertiary as well as primary prevention for the entire population. Yet, preventative efforts on the population level are rare. Current efforts in prevention focus mainly on physical activity and obesity (49). Prevention, however, can and must encompass multiple factors, including access to higher education. Education is not only important to promote a longer lifetime period with better cognitive functioning, but education also strengthens the cognitive competencies necessary for social and economic participation of the elderly population. Hence, implementing programs that encourage and support higher education must be a major focus of public policy.

**Table 1:** Some important challenges for implementing intellectual stimulation within prevention programs.

**Form of intellectual stimulation**	**Challenges**
Education	• Personal support, tutoring programs, and special programs for underachievers starting in early childhood • Psychological support and guidance for children in difficult familiar situations • Financial support for children from low socioeconomic groups for school books, additional learning material and software • Enabling access to higher education for everyone • Higher education programs focused on specific skills (talent-based) specifically for people with low academic performance
Occupational demands	• Willingness to adapt work activity schedules in low cognitively demanding jobs (e.g., rotating activities, adding more demanding tasks etc.) • Offer additional cognitive activities in between regular work tasks for people in low demand jobs • Enhanced skill development in companies and supporting workers in developing better stress management skills to meet increased demands • Government-subsidy for expanding cognitively demanding work situations • Time available to pursue cognitively stimulating activities.
Leisure activities	• Availability of offers in the area • Knowledge about relevant offers in the area (effective communication pathways) • Availability of transportation to the activity • Motivation to go to the activity • Offers that match personal preferences • Social networks fostering cognitive leisure activities • Financial means to cover the costs of the activities or government-subsidized activities • Willingness to adjust personal habits and personal goal that encompass enhancing cognitive stimulating activities
Cognitive training	• Availability of effective cognitive training tools • Knowledge of the most effective combination of training levels and types of training activities based on individual skills and needs • Adherence to cognitive training programs • Motivation to perform well (e.g., gamification component) • Access to necessary IT equipment and/or internet connectivity • Necessary IT skills • Availability of training programs that require fewer qualified workers to supervise • Qualification of staff necessary to implement training programs on a large scale

*e.g., for example; etc., et cetera meaning and so forth; IT, information technology*.

The process of translating research into policy is generally slow because the implementation of such programs requires a collaborative approach in raising awareness, reducing risk, informing local communities, targeting social inequalities, developing programs for high-risk groups, and establishing effective healthcare structures (49)—some challenges are mentioned in Table 1. Overall, it is useful to offer programs tailored to each person's individual risk profile. Personalized interventions can include social engagement strategies for the socially isolated people, nutrition plans for those with high blood sugar and high blood lipids, cognitive trainings for those with low education and those who worked in jobs with low mental demands, and others. Personalizing interventions is a more efficient use of resources than offering standardized multimodal programs. This is because the latter requires each participant to undergo the same program irrespective of individual need and characteristics, which is more expensive and stressful to the individual. Interventions for cognitive health that are tailored to the person are more likely to bring the best benefits with minimum burden. There are first tailored interventions implemented on a local level for managing behavioral disturbances of patients with frontotemporal dementia (50) and for preventing falls (51). Yet, to our knowledge, there seem to be no publicly known tailored intervention program for dementia prevention. This should be addressed urgently.

## Conclusion

Aging is our future and every one of us wants to enjoy a long and happy life. To protect our cognitive abilities in old age, we need to be active now. Prevention starts today and evidence has shown that intellectual stimulation, such as our education and what we do every day at work, can strengthen our resilience against dementia. Despite the evidence, public policy is slow in implementing relevant prevention strategies. Encouraging higher education for everyone and supporting continuous education in mid- and old-age, including cognitive stimulation with complex information processing in medical treatment plans, and developing person-tailored intervention programs are just some steps in the right direction. Given that maintaining cognitive health in old age is one of the major challenges of aging societies like ours, we urgently need to recognize the potential of non-biological risk factors across the life-course in medical treatment plans and public policy.

## Author Contributions

FR wrote the manuscript and approved of the final version to be submitted for publication.

## Conflict of Interest

The author declares that the research was conducted in the absence of any commercial or financial relationships that could be construed as a potential conflict of interest.
